# Antimicrobial susceptibility of bacterial isolates from community acquired infections in Sub-Saharan Africa and Asian low and middle income countries

**DOI:** 10.1111/j.1365-3156.2011.02822.x

**Published:** 2011-06-24

**Authors:** Elizabeth A Ashley, Yoel Lubell, Nicholas J White, Paul Turner

**Affiliations:** 1 Mahidol-Oxford Tropical Medicine Research Unit, Mahidol UniversityBangkok, Thailand; 2 Imperial College NHS TrustLondon, UK; 3 Centre for Tropical Medicine, University of OxfordOxford, UK; 4 Shoklo Malaria Research UnitMae Sot, Thailand

**Keywords:** sepsis, Africa, Asia, antibiotics, antimicrobial resistance, *Escherichia coli*, *Klebsiella* spp., *Staphylococcus aureus*, *Streptococcus pneumoniae*

## Abstract

**Objective:**

Antimicrobial resistance has arisen across the globe in both nosocomial and community settings as a consequence of widespread antibiotic consumption. Poor availability of laboratory diagnosis means that resistance frequently goes unrecognised and may only be detected as clinical treatment failure. In this review, we provide an overview of the reported susceptibility of common community acquired bacterial pathogens in Sub-Saharan Africa and Asia to the antibiotics that are most widely used in these areas.

**Methods:**

We reviewed the literature for reports of the susceptibility of prevalent pathogens in the community in SSA and Asia to a range of commonly prescribed antibiotics. Inclusion criteria required that isolates were collected since 2004 and that they were obtained from either normally sterile sites or urine. The data were aggregated by region and by age group.

**Results:**

Eighty-three studies were identified since 2004 which reported the antimicrobial susceptibilities of common bacterial pathogens. Different methods were used to assess in-vitro susceptibility in the different studies. The quality of testing (evidenced by resistance profiles) also varied considerably. For *Streptococcus pneumoniae* and *Neisseria meningitidis* most drugs maintained relatively high efficacy, apart from co-trimoxazole to which there were high levels of resistance in most of the pathogens surveyed.

**Conclusions:**

Compared with the enormous infectious disease burden and widespread use of antibiotics there are relatively few reliable data on antimicrobial susceptibility from tropical Asia and Africa upon which to draw firm conclusions, although it is evident that many commonly used antibiotics face considerable resistance in prevalent bacterial pathogens. This is likely to exacerbate morbidity and mortality. Investment in improved antimicrobial susceptibility testing and surveillance systems is likely to be a highly cost-effective strategy and should be complemented by centralized and readily accessible information resources.

## Introduction

Antimicrobial resistance hinders effective treatment of some of the leading causes of morbidity and mortality in the developing world. In resource poor settings, the effects can be particularly damaging as alternative more effective therapies are often either unaffordable or unavailable. Whilst higher level health facilities in urban areas might have the ability to detect the occurrence of resistance, particularly in nosocomial infections, in many areas in Sub-Saharan Africa (SSA) and Asia most illness episodes and their treatment will occur within the community Awareness of antimicrobial resistance is less well established and the ability to mitigate its consequences is limited in most areas of the rural tropics. Antibacterial drug resistance in community acquired infections is also less well documented than in nosocomial infections. There is an urgent need for improved surveillance.

Recent reviews of antimicrobial susceptibility in the developing world have indicated generally increasing trends in resistance amongst some of the leading infectious disease causes of morbidity and mortality (Okeke *et al.* 2005a, b; Reddy *et al.* 2010). The authors describe increasing resistance in almost all pathogens surveyed, including multidrug resistance in common bacterial infections such as those caused by *Streptococcus pneumoniae* and *Mycobacterium tuberculosis*.

In this review, we provide an updated overview of reported *in vitro* susceptibilities of community acquired bacterial pathogens to frequently used antibiotics in low and middle income countries in Sub-Saharan Africa and Asia. The antibiotics included in the review comprise the beta-lactams, including the third generation cephalosporins (cefotaxime and ceftriaxone), macrolides, chloramphenicol, cotrimoxazole and gentamicin. The pathogens we focused on were *S. pneumoniae,* Group A *Streptococcus* (GAS), Group B *Streptococcus* (GBS)*, Staphylococcus aureus, Neisseria meningitidis, Haemophilus influenzae, Escherichia coli, Klebsiella* spp*., S. typhi, S. paratyphi*, and non-typhoid salmonellae (NTS).

## Antimicrobial susceptibility testing **(**AST**)**

Antimicrobial susceptibility testing is used routinely by diagnostic microbiology laboratories to direct therapy. The ‘gold standard’ for susceptibility testing is determination of the minimum inhibitory concentration (MIC), i.e. the lowest concentration of antimicrobial that will inhibit the visible growth of a micro-organism after overnight incubation. The range of antibiotic concentrations used for determining MICs is universally accepted to be in doubling dilution steps up or down from 1 mg/l. Methods for determining the MIC include the broth microdilution method, where wells contain broth with different dilutions of antibiotics added, and agar dilution techniques that use agar into which antimicrobial agents have been incorporated at different concentrations. The E-test™ is a modified agar diffusion method in which an agar plate is inoculated with a bacterial isolate and a rectangular strip impregnated with antibiotic is overlaid; the drug diffuses out into the agar, producing an exponential gradient of drug concentrations. The MIC corresponding to the zone of inhibition is read off a scale on the strip. In practice, estimating precise MICs for various drugs against individual isolates is labour-intensive and time-consuming, so the most common method employed by most diagnostic laboratories is a simpler agar diffusion test (Kirby–Bauer method), in which the organism under investigation is inoculated onto an agar plate and exposed to a diffusion gradient of antibiotic from an impregnated disc of filter paper placed on the agar surface. The circular area of growth inhibition (zone size) reflects the antibiotic activity. This method provides a simple and cheap ‘breakpoint technique’, using zone of inhibition cut-offs to classify bacterial isolates as either susceptible, intermediate, or resistant.

There is not one universally accepted system for AST, meaning different countries/laboratories use different breakpoints to define susceptibility of different bacteria. However, since 2002 there has been an attempt to harmonize MIC breakpoints in Europe, co-ordinated by the European Committee on Antimicrobial Susceptibility Testing (EUCAST). In the USA, most laboratories follow the Clinical and Laboratory Standards Institute (CLSI) recommended methods for disc susceptibility testing (previously the National Committee for Clinical Laboratory Standards). There are further moves towards international harmonization and standardization.

AST methods have limitations. The breakpoints as defined are intended as a guide and the results are not always mirrored by the clinical response to treatment which may be better or worse than the AST results would predict. MICs reflect the relationship between extracellular organism growth and extracellular antibiotic concentrations. For example, aminoglycosides have very poor cellular penetration; organisms with intracellular pathogenesis (e.g. *Salmonellae*) may appear sensitive in-vitro but do not respond in-vivo. For certain organism–antimicrobial combinations no MIC breakpoints exist, e.g. azithromycin and the *Enterobacteriaceae*. The latest British (BSAC) guidelines state that ‘azithromycin has been used in the treatment of infections with *S. typhi* (MIC <16 mg/l for wild-type isolates) and some enteric infections’ (Andrews 2009). In fact azithromycin is an excellent treatment for quinolone resistant enteric fever, as evidenced by trials which have enrolled several hundred patients (Effa & Bukirwa 2008; Shah 2009). This is because azithromycin concentrates considerably in leukocytes and various body tissues. This has raised the question as to whether MIC breakpoints should be set based on achievable concentrations in blood or in tissue. The pharmacokinetic properties of the drug and the ability to attain adequate drug concentrations at the site of the infection are key determinants of treatment success or failure.

Despite its limitations, AST is generally regarded as providing a useful guide to local patterns of resistance. The methods described rely on phenotypic testing of the bacteria isolated from clinical samples. There is interest to develop and validate genotypic AST methods but at present, these are used mainly in reference laboratories.

As there are few functioning diagnostic microbiology laboratories outside the capital cities in most tropical countries, information from AST in rural areas is often not available. This is a major disadvantage, since before deploying an antimicrobial agent on a large scale, it is important to know its likely efficacy against local pathogens.

## Methods

The selection of pathogens to include in the review was based on the literature describing common pathogens in community acquired infections in low and middle income countries, with an emphasis on younger age groups (Mulholland *et al.* 1999; Phetsouvanh *et al.* 2006; Hill *et al.* 2007; Rudan *et al.* 2008; Zaidi *et al.* 2009).

Studies were selected using the following inclusion criteria:

- Bacterial isolates were collected from patients and subjected to antimicrobial susceptibility testing.
- The isolates were collected in a country classified by the World Bank as low or middle income and placed in one of the four WHO regions of AFRO, SEARO, WPRO or EMRO.
- Infections were community acquired as classified in the study or inferred by context.
- Isolates were collected from 2004 onwards. Where studies contained data from earlier years as well, these data were excluded if reported separately, otherwise the study was included. A small number of exceptions were made where a study was considered particularly relevant and where no other data were available (Phetsouvanh *et al.* 2006; Morrissey *et al.* 2007; Ochiai *et al.* 2008).
- Isolates were obtained from individuals of any age over 1 month. Resistance in isolates from neonates are described in a separate review as the prevalence of different pathogens and their susceptibility patterns are expected to differ (Lubell *et al.* 2011).
- Isolates were from either normally sterile sites or urine. Urinary isolates were included to maximize the information obtained on the prevalence of extended spectrum beta lactamase (ESBL) producing organisms.

The following search terms were used to search Pubmed, Scopus and Embase: (ceftria* one OR cefotaxime OR ceftazidime OR cephalosporin OR ampicillin OR amoxicillin OR aminopenicillin OR cotrimoxazole OR co-trimoxazole OR gentam* OR trimethoprim OR chloramphenicol OR azithrom* OR clarithrom* OR erythrom* OR macrolide OR lactam); country names for all low and middle income countries in SSA and Asia; klebsiella OR agalactiae OR ‘group B streptococci’ OR Streptococc* OR pneumo* OR ‘Group A streptococci’ OR pyogenes OR aureus OR Salmonell* OR enteritidis OR typhi* OR coli OR mening* OR influenz; studies published in English in the previous 5 years. The last search was conducted on 25th June 2009.

The total number of isolates tested and the number of susceptible isolates were recorded in a database along with other study characteristics, including the region, population age-group, whether the isolate source was a normally sterile site, and susceptibility testing method. Where MIC90 (minimum inhibitory concentration required to inhibit the growth of 90% of organisms) was reported, this was also recorded. No attempt was made to harmonize breakpoints. For studies reporting on *S. aureus* susceptibility to any of the drugs, data on susceptibility to methicillin, oxacillin, cloxacillin or flucloxacillin were also collected to infer the proportion of methicillin-resistant *Staphylococcus aureus* (MRSA) isolates. Where only one or two studies were available for a particular pathogen, the findings are summarized individually. Where more than two studies were found for a pathogen, a meta-analysis using a random-effects model and the DerSimonian and Laird method was used to estimate the pooled susceptibility, along with sub-group analysis for region and age (Dersimonian & Kacker 2007). Multivariable regression was also used to estimate the effect of these covariates. The analysis was carried out with STATA 10 (StataCorp, College Station, TX, USA).

## Results

The initial search identified 562 potentially relevant papers. Abstract and main text scanning ruled out 486 of these. Reasons for exclusion were primarily: (i) recruitment of nosocomial infections; (ii) isolates collected earlier than 2004; (iii) isolates were obtained from normally non-sterile sites; (iv) pathogens were undifferentiated (either in entirety or as Gram positive/negative); (v) the study population were neonates. Seven further studies were identified by searching references and consulting experts in the field. A total of 83 relevant studies were included in the final review, 32 of which obtained their isolates in SSA and 51 in Asia.

Just over half the studies included patients of all ages; 24 papers included patients under 5 years, 10 included children over 5, and 7 included adults (the age-groups did not always correspond to this grouping, therefore in some instances the classification had to be inferred, e.g. papers that focused on school children were included in the children over 5 age group). The number of papers containing susceptibility data for each of the pathogens classified by geographical region and age is shown in Table 1. For Group A *Streptococcus* no data were available and it was therefore excluded from the results.

**Table 1:** Number of studies reporting on each of the pathogens by continent and age

Pathogen	All	SSA	Asia	<5 y/o	5–15 y	Adults	Unspecified
*S. pneumoniae*	29	18	11	17	5	0	7
*S. aureus*	15	8	7	4	3	2	6
GBS	1	1	0	0	1	0	0
*N. meningitidis*	10	4	6	0	5	0	5
*Klebsiella* spp.	17	7	10	3	3	4	7
*S. typhi*	23	2	21	1	0	1	21
*S. paratyphi*	11	0	11	0	0	1	10
*E. coli*	22	10	12	4	3	6	9
NTS	10	8	2	4	2	0	4
*H. influenzae*	11	4	7	6	4	0	1

NTS, non-typhoidal Salmonellae; GBS, Group B streptococci.

### Antibiotic resistance in Gram-positive pathogens

#### S. pneumoniae

The largest number of studies evaluated susceptibility of *S. pneumoniae* isolates. However, interpretation of susceptibility data for *S. pneumoniae* is complicated by several factors. Disc testing is not recommended for assessing susceptibility to several drugs, including amoxicillin/ampicillin (the aminopenicillins) and the cephalosporins. Susceptibility to these beta-lactams can be inferred in isolates that are susceptible to penicillin G (either on the basis of an oxacillin disc zone diameter of ≥20 mm or an MIC of <1 mg/l). In penicillin non-susceptible isolates, MICs should be determined for the beta-lactam drugs. Finally, CLSI beta-lactam breakpoints have recently been revised reflecting clinical observations of penicillin treatment success in traditionally non-susceptible isolates (penicillin MIC ≥1 mg/l). We found considerable heterogeneity in pneumococcal susceptibility testing between studies and MIC breakpoints were often not explicitly stated. Therefore we inferred beta-lactam (penicillin and amoxicillin/ampicillin) susceptibility on the basis of the penicillin result: either a sensitive disc test result (assumed to be 1 μg oxacillin disc zone of at least 20 mm: although more often than not this was not stated) or a reported ‘susceptible’ MIC category, unless specific results for amoxicillin/ampicillin or cefotaxime/ceftriaxone were given (in which cases we used these).

**Figure 1:**
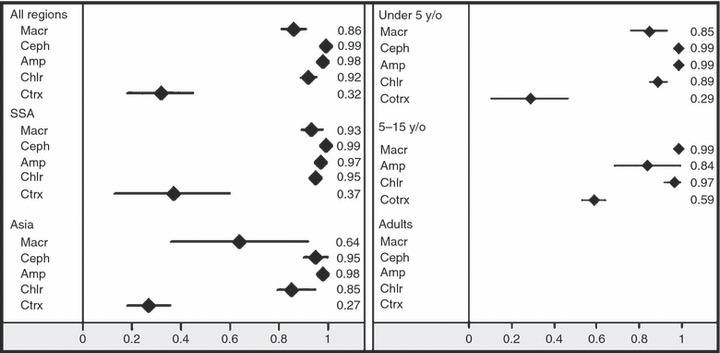
Proportion susceptible of *S. pneumoniae* by region (left) and age group (right). Macr – Macrolides; Ceph –**third** generation cephalosporins; Amp – aminopenicillins; Chlr – chloramphenicol; Ctrx – co-trimoxazole; Gent – gentamicin.

High levels of resistance were found only to co-trimoxazole, where the weighted mean proportion of susceptible isolates was 0.32 (95% CI 0.18–0.46; random effects model). The most effective antibiotics based on *in vitro* testing were amoxicillin/ampicillin and the third generation cephalosporins (Figure 1).

The single largest study in the dataset came from South Africa. This reported *S. pneumoniae* susceptibility to macrolides from the year 2000 to 2005, and showed a gradual increase in non-susceptibility from 9% to 14% in 2005 (only data for 2004/2005 are included in this review).

#### S. aureus

**Figure 2:**
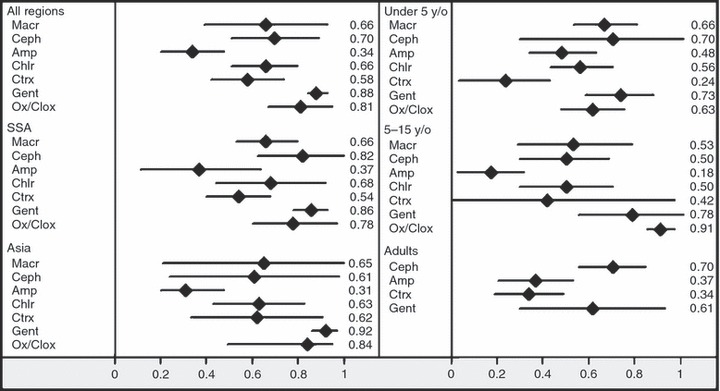
Proportion susceptible of *S. aureus* by region (left) and age group (right). Macr – macrolides; Ceph –**third** generation cephalosporins; Amp – aminopenicillins; Chlr – chloramphenicol; Ctrx – co-trimoxazole; Gent – gentamicin; Ox/Clox – oxacillin/cloxacillin.

Overall gentamicin was the most consistently active antibiotic against *S. aureus* in both SSA and Asia (Figure 2), although it is generally not recommended for mono-therapy in Gram positive organisms. Gentamicin is often empirically prescribed for sepsis along with ampicillin which has no anti-staphylococcal activity and where *S. aureus* is a potential pathogen (WHO 2005). *S. aureus* showed a relatively high degree of resistance to the other surveyed drugs, and to amoxicillin/ampicillin in particular. Results were widely dispersed for macrolides in Asia and co-trimoxazole in both regions. For amoxicillin/ampicillin and chloramphenicol results were mostly consistent, with the range of results from individual studies reflected in the uncertainty intervals in Figure 2. But two studies found relatively high levels of susceptibility to chloramphenicol in Nepal and Thailand (Phetsouvanh *et al.* 2006; Nickerson *et al.* 2009).

Seven of the 15 studies reporting on *S. aureus* susceptibility to the other drugs included data on susceptibility to methicillin, oxacillin, cloxacillin or flucoxacillin; the overall estimate for MRSA from these studies is 19% in the community, with no significant difference in regions (Bachou *et al.* 2006; Brink *et al.* 2007; Hill *et al.* 2007; Farajnia *et al.* 2009; Nickerson *et al.* 2009; Onipede *et al.* 2009; Sigauque *et al.* 2009). While for almost all other pathogens third generation cephalosporins showed consistently high efficacy, *S. aureus* exhibited considerable variation in susceptibility in individual Asian study results. This is likely to indicate either a problem of susceptibility testing, or more likely that a significant proportion of these isolates were in fact MRSA. The two studies that included data on oxacillin/cloxacillin and ceftriaxone showed similar levels of susceptibility (Bachou *et al.* 2006; Onipede *et al.* 2009). The single largest study was from Asia where only 10 of the 40 isolates were found to be susceptible to ceftriaxone (Asghar *et al.* 2008). In Africa susceptibility levels were a little higher, although the difference was not significant. The two largest studies from SSA both showed that approximately two-thirds of *S. aureus* isolates were susceptible to ceftriaxone (Akinloye *et al.* 2006; Bachou *et al.* 2006).

#### Group B streptococci

Only one study reported on the susceptibility of GBS, showing that amongst children with community acquired bacteraemia, GBS accounted for 20% of identified blood stream infections in a paediatric ward in Mozambique (Sigauque *et al.* 2009). Only 4 of the 33 isolates were from infants over 1 month and there was no breakdown of the susceptibility by age group; all isolates, however, were susceptible to amoxicillin/ampicillin. The proportion of isolates Susceptible to chloramphenicol was 0.71 (95% CI 0.56–0.86) and to co-trimoxazole 0.85 (0.73–0.97) for all isolates.

### Antibiotic resistance in Gram-negative pathogens

#### N. meningitidis

**Figure 3:**
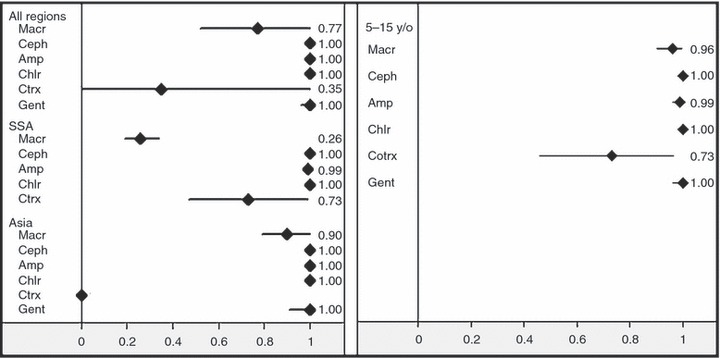
Proportion susceptible of *N. meningitidis* by region (left) and age group (right). Macr – macrolides; Ceph –**third** generation cephalosporins; Amp – aminopenicillins; Chlr – chloramphenicol; Ctrx – co-trimoxazole; Gent – gentamicin.

All drugs maintained good efficacy against *N. meningitidis* with the exception of the macrolides and co-trimoxazole (Figure 3). For the macrolides the only available study from SSA was a multi-centre investigation, which found high levels of resistance to macrolides across the 16 countries from which data were obtained (Hedberg *et al.* 2009). All three studies from Asia on the other hand showed good macrolide efficacy (Kumar *et al.* 2008; Hossain *et al.* 2009; Jhamb *et al.* 2009; Nair *et al.* 2009).

For co-trimoxazole only two studies were available, one from SSA and one from Asia. The study by Sigauque *et al.* (2009) found 8 of the 11 bacteraemias caused by *N. meningitidis* to be susceptible to co-trimoxazole in Mozambique (Sigauque *et al.* 2009). Hossain *et al.* (2009) in Dhaka, Bangladesh, showed increasing resistance to co-trimoxazole from 50% in 2002 to 100% in 2006 (only data from 2004 onwards were included in this review, by which time all isolates were resistant to co-trimoxazole) (Hossain *et al.* 2009).

#### H. influenzae

**Figure 4:**
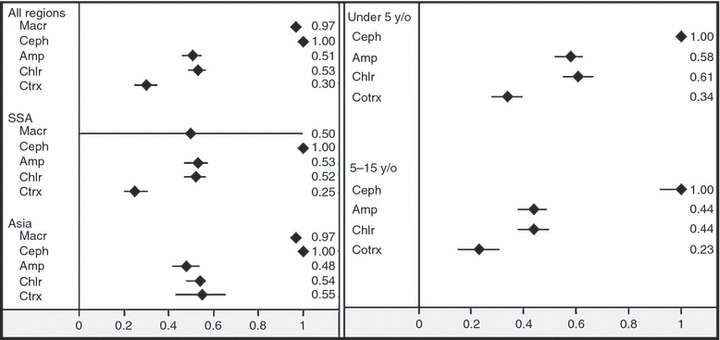
Proportion susceptible of *H. influenzae* by region (left) and age group (right). Macr – macrolides; Ceph –**third** generation cephalosporins; Amp – aminopenicillins; Chlr – chloramphenicol; Ctrx – co-trimoxazole; Gent – gentamicin.

For *H. influenzae* only the macrolides and third generation cephalosporins maintained high levels of susceptibility (Figure 4), although almost no data were available from SSA to support macrolides, while for the cephalosporins more studies were able to validate their susceptibility. Only one of these papers reported on quinolone susceptibility; there was no resistance in the 73 isolates tested (Rahman *et al.* 2008).

#### *Klebsiella* spp

**Figure 5:**
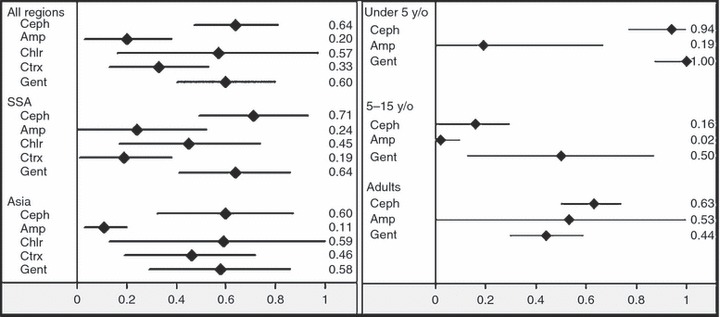
Proportion susceptible of *Klebsiella* spp. by region (left) and age group (right). Macr – macrolides; Ceph –**third** generation cephalosporins; Amp – aminopenicillins; Chlr – chloramphenicol; Ctrx – co-trimoxazole; Gent – gentamicin.

There were relatively high rates of resistance in *Klebsiella* spp. to all the surveyed antibiotics, and particularly to amoxcillin/ampicillin, as would be expected, and to co-trimoxazole, with estimated susceptibility levels of 20% and 33%, respectively (Figure 5).

Only two studies reported susceptibility of *Klebsiella* spp. to macrolides, one of which found the proportion of isolates susceptible to azithromycin to be 0.64 (95% CI 0.5–0.78) (Chayani *et al.* 2009), while in the second study of children with osteomyelitis in Iran, none of the four isolates were susceptible to erythromycin (Mirnejad *et al.* 2008).

For third generation cephalosporins, results indicated high resistance rates in the 5–15 year old age group as compared with younger children and adults, although this sub-group included only one study with four synovial fluid isolates from Iran (Mirnejad *et al.* 2008) and one study from India surveying children with bacterial meningitis (Shameem *et al.* 2008). These same two studies also found low susceptibility to chloramphenicol with none of the four isolates in the Iranian study and three of the eight isolates in the Indian study being susceptible.

#### E. coli

**Figure 6:**
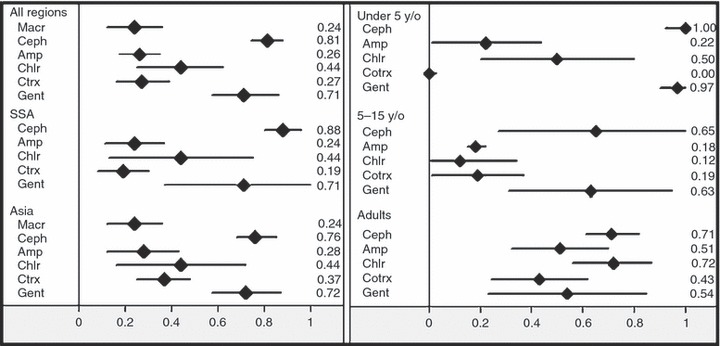
Proportion susceptible of *E. coli* by region (left) and age group (right). Macr – macrolides; Ceph –**third** generation cephalosporins; Amp – aminopenicillins; Chlr – chloramphenicol; Ctrx – co-trimoxazole; Gent – gentamicin.

A relatively large number of studies reported on *E. coli* susceptibility, with amoxicillin/ampicillin, chloramphenicol, co-trimoxazole all facing high levels of resistance in both SSA and Asia (Figure 6). For the macrolides, which are not usually used for treatment of *E. coli* infection, only two studies were available, both based in Asia. Chayani *et al.* (2009) report only 19 of 96 susceptible isolates and in Khan and Zaman (2006) 31 of 102 isolates were susceptible to erythromycin. Gentamicin susceptibility was reduced in both African and Asian *E. coli* isolates, at around 70%. Identification of extended spectrum beta lactamase producing *Enterobacteriaceae* was not reported.

#### *S. typhi* and *S. paratyphi*

**Figure 7:**
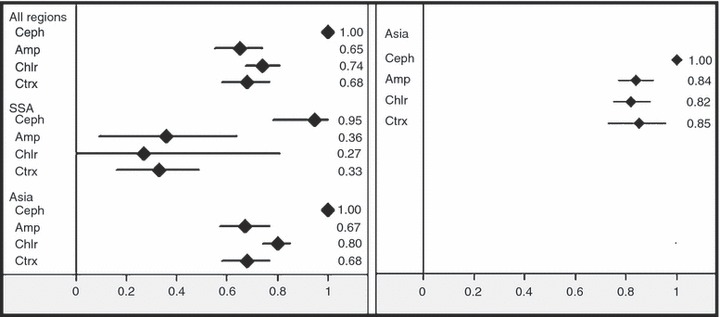
Proportion susceptible *S. typhi* (left) and *S. paratyphi* (right) by region. Macr – macrolides; Ceph –**third** generation cephalosporins; Amp – aminopenicillins; Chlr – chloramphenicol; Ctrx – co-trimoxazole; Gent – gentamicin.

Third generation cephalosporins performed well against *S. typhi* and *S. paratyphi* in all available studies (Figure 7). Amoxicillin/ampicillin, chloramphenicol and co-trimoxazole were less effective and particularly so in SSA, although the data from Africa were obtained from only two studies with 5 and 29 *S. typhi* isolates each and no *S. paratyphi* isolates.

Given the lack of consensus on relevant breakpoints, the two studies that reported susceptibility of *S. typhi* and *S. paratyphi* to macrolides reported their results in terms of the MIC range and MIC90, rather than susceptibility rates. Dolocek *et al.* (2008) reported a range of 4–16 mg/l and a MIC90 of 12 mg/l for azithromycin amongst the 126 *S. typhi* isolates, while all five *S. paratyphi* isolates were classified as susceptible to azithromycin. These results were correlated with good clinical efficacy. Capoor *et al.* (2009) report a MIC90 of 8 mg/l amongst 149 *S. typhi* isolates and a MIC90 of 4 mg/l in the 43 *S. paratyphi* isolates.

#### Non-Typhoidal Salmonellae (NTS)

**Figure 8:**
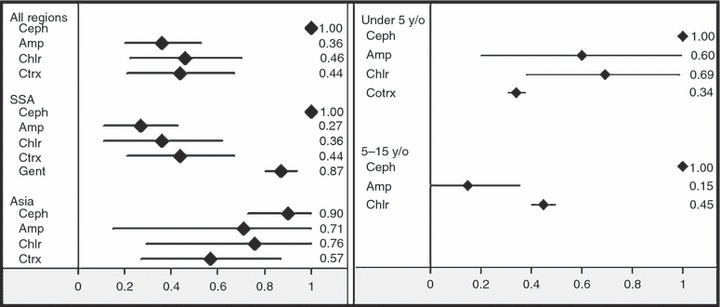
Proportion susceptible of non-typhoid salmonellae by region (left) and age group (right). Macr – macrolides; Ceph –**third** generation cephalosporins; Amp – aminopenicillins; Chlr – chloramphenicol; Ctrx – co trimoxazole.

**Figure 9:**
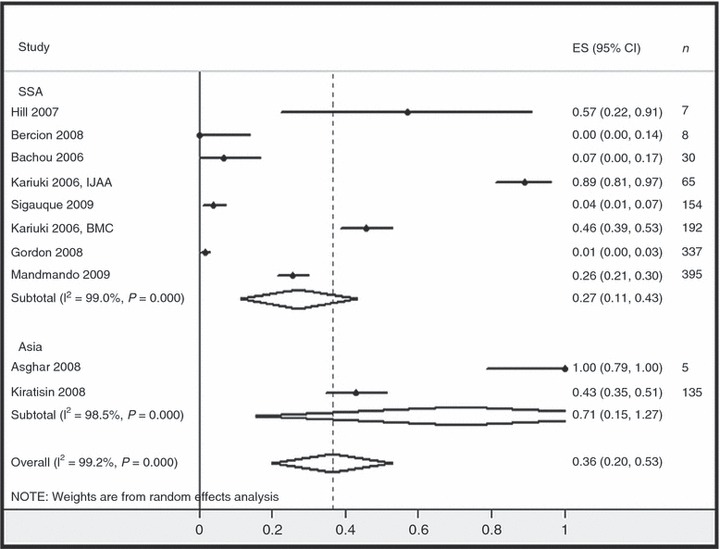
Individual studies reporting the susceptibility of non-typhoid salmonellae to aminopenicillins. *n*– number of isolates in study.

NTS were most susceptible to third generation cephalosporins across both regions and age groups (Figure 8). For gentamicin results in SSA were between 0.82 and 1 for all four studies (Bachou *et al.* 2006; Hill *et al.* 2007; Mandomando *et al.* 2009; Sigauque *et al.* 2009). For amoxicillin/ampicillin results were particularly diverse (Figure 9) although the larger studies in SSA mostly found very high levels of resistance in NTS.

## Discussion

This review summarizes the current state of our knowledge regarding antimicrobial susceptibility of the common organisms causing serious community-acquired infections in low income countries. It demonstrates that little data exist to inform empiric treatment guidelines in these countries and that there are high levels of resistance to many commonly used antibiotics. It is also clear that significant variations exists between and within regions and countries, such as the higher proportion of macrolide non-susceptible pneumococci in Asia. As only a tiny fraction of treated bacterial infections have culture and susceptibility testing, resistance patterns are most useful in determining the policies and choices of empirical antibiotic treatment.

*S*. *pneumoniae* is probably the most important pathogen in terms of associated mortality (O’brien *et al.* 2009), and the pathogen on which vaccine development efforts have concentrated. Globally pneumococcal resistance to antimicrobials is rising. This does not seem to have been mitigated by the introduction of the conjugate vaccine in developed countries as was hoped initially, although the vaccine has not yet been deployed in most countries included in this review. This review suggests that in developing countries, the most frequently-used empiric antimicrobials retain efficacy with the exception of co-trimoxazole and, in Asia, the macrolides. *Streptococcus pneumoniae* illustrates the need for appropriate (i.e. clinically guided) MIC breakpoints. Prior to 2008, pneumococci with MICs of >0.06 mg/l were considered non-susceptible to penicillin, but it was observed that patients with non-meningeal pneumococcal infections were usually cured of their infection by penicillin. More recent CLSI guidelines reflect this clinical observation and the breakpoints for non-meningeal pneumococcal infection have been adjusted (now MICs of ≤2 mg/l are considered penicillin susceptible) (Weinstein *et al.* 2009). Likewise the activity of azithromycin *in vitro* may not be a good indication of its clinical performance because of its marked intracellular accumulation.

Methicillin resistance in community acquired *Staphylococcus aureus* infections is now widespread, and found even in the rural areas of low income countries. This has been noted in studies from Asia (Deleo *et al.* 2000; Chheng *et al.* 2009; Nickerson *et al.* 2009) and is also evident in Africa as indicated by a number of studies in this review (Bachou *et al.* 2006; Brink *et al.* 2007; Hill *et al.* 2007; Onipede *et al.* 2009; Sigauque *et al.* 2009). This methicillin resistance compromises the efficacy of beta-lactam antibiotics. Oral agents such as clindamycin and linezolid are often unaffordable and parenteral agents such as vancomycin are often unavailable. Interestingly, doxycycline and co-trimoxazole are usually effective – but neither would be a first choice empirical treatment.

Of considerable concern is increasing resistance in the *Enterobacteriaceae.* Our data reveal that affordable first-line agents such as ampicillin and gentamicin are unlikely to be clinically efficacious in a substantial proportion of infections. This results in increasing reliance on the third generation cephalosporins for empirical treatment of serious infections. However, the spread of extended-spectrum beta-lactamase producing strains into the community, probably accelerated by this increased consumption, is eroding the usefulness of these drugs. Alternative agents for treating multi-resistant coliform infections, such as the carbapenems, are unaffordable for treatment of community-acquired infections in low-income countries. Fluoroquinolone resistance in typhoid in Asia has severely curtailed the usefulness of these drugs. In Africa, where NTS are of greater importance there have been no clinical trials of fluoroquinolones. As quinolone-resistant salmonellae infections become more common, an alternative oral antimicrobial is required for settings where parenteral ceftriaxone is not a treatment option. Azithromycin is clearly an excellent drug for these infections, but laboratory data to support clinical trial data are lacking.

## Limitations

The comparison of results across studies is problematic since different AST methods and breakpoints have been used. This is highlighted by the results of AST testing in *S. pneumoniae*. In this review the proportion of susceptible isolates was summarized as reported in the studies and not reinterpreted with standardized breakpoints as most studies did not provide the MICs for all isolates. Given the variation in breakpoint guidelines, together with the different AST methods used, variability in results is not unexpected. Nevertheless, the reliability and quality of reporting of some of the results is questionable. For example in one study a large proportion of *S. aureus* isolates were reported as being cefoxitin resistant and yet were not identified as MRSA. In other studies third generation cephalosporin resistance in coliforms did not lead to confirmatory tests for ESBL production being performed. Another group reported all Gram negative *Enterobacter* spp. isolates as being susceptible and a large proportion of *S. aureus* isolates as being resistant to vancomycin both of which are implausible. Finally, one study reported 24% of pneumococcal isolates to be penicillin resistant by disc testing (which may be unreliable) but did not confirm this by formal MIC testing as is recommended.

Identification methods were not always clearly specified in studies reporting multidrug resistant *S. aureus*. It is possible for isolates to be misclassified (i.e. for the staphylococci to be identified as *S. aureus*). It is also reasonable to expect considerable heterogeneity in MRSA prevalence within countries. Nevertheless, the indication of relatively high levels of MRSA in the community suggested by these studies is an area requiring urgent surveillance and research.

Aside from methodological concerns, pooling susceptibility data from different studies is also problematic because of genuine geographical heterogeneity which might not warrant their aggregation, as a larger study in one area can heavily discount equally valid information from other areas with genuinely different levels of resistance. For *S. pneumoniae*, the study with the lowest susceptibility levels (Morrissey *et al.* 2007) and the single largest study, including over 7000 isolates (Wolter *et al.* 2008), were from South Africa, which might be less representative of other areas in SSA. Use of a random effects model, however, implies that the effect of the trial size is mitigated to allow for between site variance. Such regional variation in antimicrobial susceptibility is to be expected. This has been demonstrated by large international studies of resistance in key pathogens, for example the PROTEKT and SENTRY projects (Hoban *et al.* 2001; Felmingham 2002; Felmingham *et al.* 2007). Both projects have focused on community-acquired infections and have a worldwide distribution of study sites, but unfortunately provide little data for large areas of the developing world.

Another possible bias is that sites with microbiological facilities are often located in urban areas and serve higher income populations, which may not be representative of much of the population in poorer areas of SSA and Asia.

## Conclusions

Antibiotic use continues to be poorly controlled in human and animal medicine in the developing world. Self medication, poor adherence to complete antibiotic regimens and low quality, often counterfeit drugs are all common. Antibiotic resistance is likely to continue to increase. More information is urgently needed from economically poor countries where the disease burden from bacterial pathogens is greatest. Investment in obtaining antimicrobial susceptibility data to inform empirical treatment guidelines would seem highly cost-effective given that many of the preventable deaths are caused by relatively few bacterial pathogens. However, maintaining a diagnostic laboratory capable of culturing organisms and performing AST in remote areas is difficult and may be unaffordable in poor settings. If it is not feasible to set up laboratories capable of doing susceptibility testing locally, isolates could be tested in centralized reference laboratories. This may result in biased sampling as organisms such as *S. pneumoniae* require careful handling in the laboratory and do not survive for prolonged periods.

While reviews such as this can assist in summarizing what data are available and highlighting the gaps in knowledge that require most urgent attention, there is a clear need for universally standardized AST methods and breakpoints to ensure conformity of results. A centralized database, similar to the WWARN initiative for tracking antimalarial resistance, that can be regularly updated to inform on geographical and longitudinal trends in antibiotic resistance could also be a valuable tool for clinicians and policy makers (Guerin *et al.* 2009).
